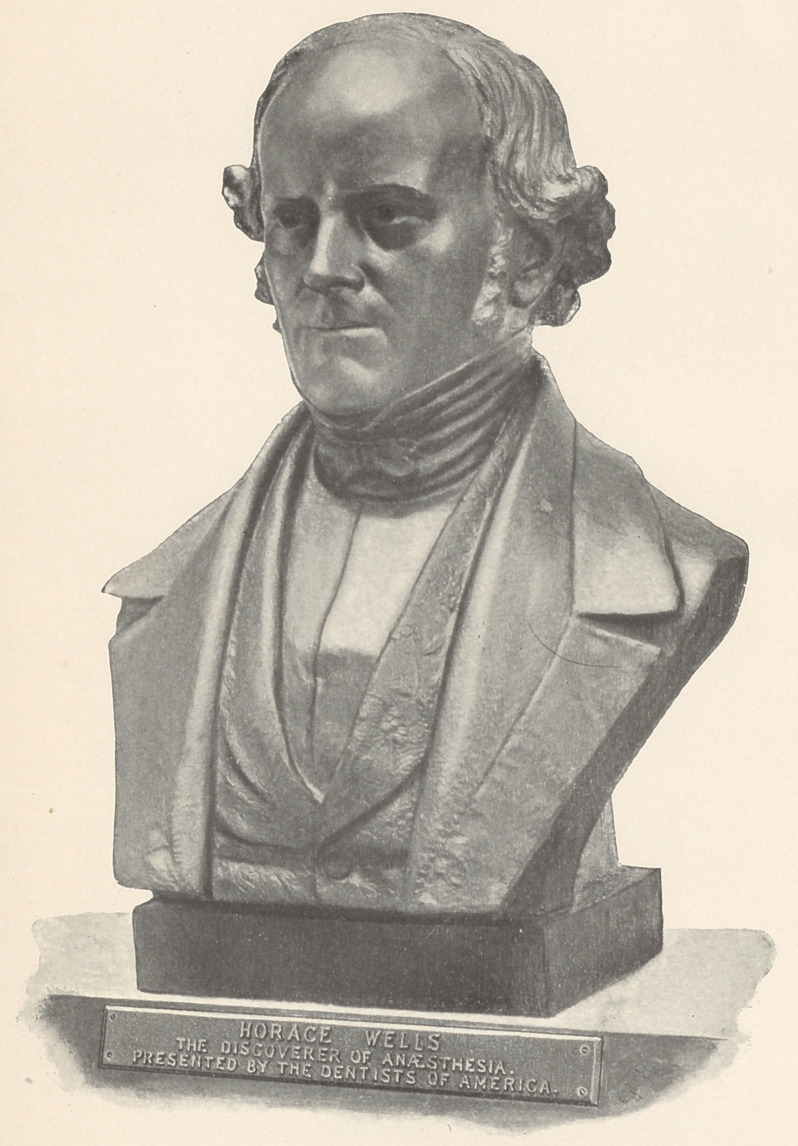# Horace Wells

**Published:** 1899-12

**Authors:** 


					﻿HORACE WELLS.
The half-tone picture which is placed as a frontispiece to this
number is an excellent reproduction of the memorial bust of this
distinguished man.
A committee having its preparation in charge was appointed
by the American Dental Association, and the funds necessary were
contributed by that organization and, through subscription, by
members of the dental profession in the United States. The work
has required time for its completion, but the result has been very
satisfactory to the committee.
The bust was modelled by J. Scott Hartley, the celebrated
sculptor of New York City, and then cast in bronze by the Gorham
Company, through the firm of Baily, Banks & Biddle, of Phila-
delphia. It is regarded by the son of Dr. Wells as being an excel-
lent likeness.
The bust will be deposited in the National Medical Museum,
Washington, D. C., by the chairman of the committee, Dr. J. D.
Thomas, to whom the dental profession owes a debt of gratitude
for his energetic efforts to have this memorial worthy the man
and the discovery it represents. Its final resting place is pecu-
liarly appropriate, and it will there remain for future generations
to honor the discoverer of anaesthesia and be an ever-present and
enduring evidence of the gratitude felt for Horace Wells by the
dental profession.
				

## Figures and Tables

**Figure f1:**